# Detection of epithelial apoptosis in pelvic ileal pouches for ulcerative colitis and familial adenomatous polyposis

**DOI:** 10.1186/1479-5876-8-11

**Published:** 2010-01-29

**Authors:** Raquel F Leal, Maria de Lourdes S Ayrizono, Marciane Milanski, João J Fagundes, Juliana C Moraes, Luciana R Meirelles, Lício A Velloso, Cláudio SR Coy

**Affiliations:** 1 Coloproctology Unit of the Surgery Department, University of Campinas (UNICAMP), Medical School, São Paulo, Brazil; 2 Internal Medicine Department, Cellular Signalization Laboratory, University of Campinas (UNICAMP), Medical School, São Paulo, Brazil; 3 Department of Pathology, University of Campinas, Medical School, Sao Paulo, Brazil

## Abstract

**Background:**

Ileal pouch-anal anastomosis (IPAA) is the surgical procedure of choice for patients with refractory ulcerative colitis (UC) and for familial adenomatous polyposis (FAP) with many rectal polyps. Pouchitis is one of the more frequent complications after IPAA in UC patients; however, it is rare in FAP.

**Objective:**

Evaluate pro-apoptotic activity in endoscopically and histological normal mucosa of the ileal pouch in patients with UC and FAP.

**Methods:**

Eighteen patients (nine with UC and nine with FAP) with J pouch after total rectocolectomy were studied. Biopsies were obtained from the mucosa of the pouch and from normal ileum. The specimens were snap-frozen and the expressions of Bax and Bcl-2 were determined by immunoblot of protein extracts and by immunohistochemistry analysis. FADD, Caspase-8, APAF-1 and Caspase-9 were evaluated by immunoprecipitation and immunoblot.

**Results:**

Patients with UC had significantly higher protein levels of Bax and APAF-1, Caspase-9 than patients with FAP, but were similar to controls. The expressions of Bcl-2 and FADD, Caspase-8 were similar in the groups. Immunohistochemistry for Bax showed less intensity of immunoreactions in FAP than in UC and Controls. Bcl-2 immunostaining was similar among the groups.

**Conclusion:**

Patients with FAP present lower levels of pro-apoptotic proteins in all methods applied, even in the absence of clinical and endoscopic pouchitis and dysplasia in the histological analysis. These findings may explain a tendency of up-regulation of apoptosis in UC patients, resulting in higher rates of progression to pouchitis in these patients, which could correlate with mucosal atrophy that occurs in inflamed tissue. However, FAP patients had low pro-apoptotic activity in the mucosa, and it could explain the tendency to low cell turn over and presence of adenomas in this syndrome.

## Backgroud

Restorative rectocolectomy with ileal pouch and anal anastomosis (IPAA) has become the surgical procedure of choice for ulcerative colitis (UC) and for familial adenomatous polyposis (FAP), for three decades [1-3]. Despite of its innumerous advantages over other therapeutic procedures, restorative retocolectomy with ileal pouch may evolve with pouchitis, a complication that affects up to 50% of patients with UC, and only 5% of patients with FAP [4-6].

Although pouchitis is a commonly reported complication, its etiology remains unknown [7-11]. Due to this difference in the incidence of pouchitis, some authors have proposed that the reactivation of UC may have a role in the induction of the local inflammation and in the increased epithelial apoptosis that will, ultimately, lead to the installation of this complication [12,13]. This hypothesis is further boosted by the fact that some patients with pouchitis have resurgence of extra-intestinal manifestations of UC in the same way as patients who have active UC [14,15], and by data supporting a goal for increased apoptosis that occurs in active UC mucosa [16,17]. The study of intrinsic and extrinsic apoptosis pathways in ileal pouch remains not completely available and there are few studies in the literature that have evaluated this putative role of pouchitis etiology.

Therefore, in order to compare the apoptotic activity in asymptomatic pouches between the highly pouchitis-prone UC patients and the pouchitis-protected patients with FAP we employed immunoblotting, immunoprecipitation assays and histological analysis to determine the expression of pro-apoptotic and anti-apoptotic proteins, and detection of apoptosis by Annexin V fluorescence microscopy in ileal pouch biopsies.

## Methods

Mucosal biopsies were taken from nine patients with non-inflamed IPAA after rectocolectomy for UC [median age 48.7 (range, 31-63) years; male 44.4%; female 55.6%], and nine patients with non-inflamed IPAA after rectocolectomy for FAP [median age 33.8 (range, 21-59) years; male 44.4%; female 55.6%]. The follow-up after the operation was 73.1 (24-168) months. The reservoir design was of the "J" type in all patients, and the right colon vascular arcade was preserved as a supplementary blood supply to the terminal ileum [18]. Mucosectomy was performed, with hand-sewn ileo-anal anastomosis. The patients had had their ileostomy closed for more than one year, at the time of the study. The absence of pouchitis was defined clinically, histology and endoscopically, according to the PDAI [19]. The control group was composed of nine individuals with normal colonoscopy examination, with a median age of 40.9 (range, 26 - 58) years and 55.6% were female. Six biopsies of each patient were obtained from terminal ileum (control) and from ileal pouch (UC and FAP).

The study was performed in accordance with the Declaration of Helsinki and was approved by the local ethical committee. All biopsies were taken after informed consent from the patients. The study was carried out at the State University of Campinas, Coloproctology Unit, and at the Cell Signaling Laboratory of the Department of Internal Medicine.

### • Immunoblotting - Gel electrophoresis

Mucosal biopsies from the pouches and from normal ileum were snap-frozen in liquid nitrogen and stored at -80°C until use. For total protein extract preparation, the fragments were homogenized in solubilization buffer at 4°C [1% Triton X-100, 100 mM Tris-HCl (pH 7.4), 100 mM sodium pyrophosphate, 100 mM sodium fluoride, 10 mM EDTA, 10 mM sodium orthovanadate, 2.0 mM phenylmethylsulfonyl fluoride (PMSF), and 0.1 mg aprotinin/ml] with a Polytron PTA 20S generator (model PT 10/35; Brinkmann Instruments, Westbury, NY) operated at maximum speed for 30 sec. Insoluble material was removed by centrifugation (20 min at 9000 × *g *at 4°C). The protein concentrations of the supernatants were determined by the Bradford dye binding method [20]. Aliquots of the resulting supernatants containing 100 μg total proteins were separated by SDS-PAGE, transferred to nitrocellulose membranes and blotted with anti-Bax, anti-Bcl-2 antibodies [21,22]. In immunoprecipitation experiments, samples containing 1.0 mg protein were incubated overnight with antibodies against FADD and APAF-1. The immunocomplexes were recovered with Protein A Sepharose, separated by SDS-PAGE, transferred to nitrocellulose membranes, and blotted with anti-Caspase-8 to FADD (extrinsic pathway apoptosis), and anti-Caspase-9 to APAF-1 antibodies (intrinsic pathway apoptosis) [21].

Reagents for SDS-PAGE, immunoblotting and immunoprecipitation were from Bio-Rad Laboratories (Richmond, CA). Phenylmethylsulfonyl fluoride, aprotinin, Triton X-100, Tween 20, glycerol were from Sigma (St. Louis, MO). Protein A-Sepharose 6 MB was from Pharmacia (Uppsala, Sweden), and nitrocellulose paper (BA85, 0.2 μm) was from Amersham (Aylesbury, UK). The anti-Bax (sc-493, rabbit polyclonal), anti-Bcl-2 (sc-492, rabbit polyclonal), anti-FADD (sc-5559, rabbit polyclonal), anti-Caspase-8 (sc-7890, rabbit polyclonal), anti-APAF-1 (sc 26685, goat polyclonal) and anti-Caspase-9 (sc-7885, rabbit polyclonal) antibodies were purchased from Santa Cruz Biotechnology, Inc. (Santa Cruz, CA). The signal was detected by chemiluminescent reaction (SuperSignal^® ^West Pico Chemiluminescent Substrate from Pierce Biothecnology, Inc. Rockford).

All numerical results are expressed as the mean ± SEM of the indicated number of experiments. The results of blots are presented as direct comparisons of bands in autoradiographs and quantified by densitometry using the Gel-Pro Analyzer 3.1 software (Exon-Intron Inc., Farrell, MD). Data were analyzed by repeat-measure ANOVA (one-way or two-way ANOVA) followed by analysis of significance (Tukey-Kramer Multiple Comparisons test), comparing UC, FAP, and control groups. The level of significance was set at p < 0.05.

### • Bax and Bcl-2 Immunohistochemistry

For immunostaining procedures, endogenous peroxidase was blocked with 3% hydrogen peroxide/10 mM PBS pH 6.0 for 15 min. Afterwards, the sections were microwaved in 3% milk buffer for 30 min and incubated overnight with primary antibody either to Bax or Bcl-2 (DAKO A/S Denmark; A3533, rabbit polyclonal and M0887, mouse polyclonal) applied in 1:500 and 1:150 dilution respectively at 20°C. The sections were incubated with post primary block and polymer secondary antibodies (Novocastra™ Laboratories Ltd; Novolink RE 7260-K) for 1 h, and processed for DAB reaction, 0.5 mg/ml (Sigma, USA, St Louis). Any cell type showing cytoplasmic staining was considered positive for qualitative analysis [23,24].

## Results

Patients with UC had significantly higher levels of Bax, APAF-1 and Caspase-9 than FAP (p < 0.05), but were similar to controls (p > 0.05). The comparison of local levels of Bcl-2 in pouches from UC, FAP patients and controls revealed that they were similar among the groups (p > 0.05).

The expression of FADD and Caspase-8 was similar among the groups (p < 0.05), however there was a tendency of high levels in UC patients when compared to other groups (p = 0.08).

**Figure 1:**
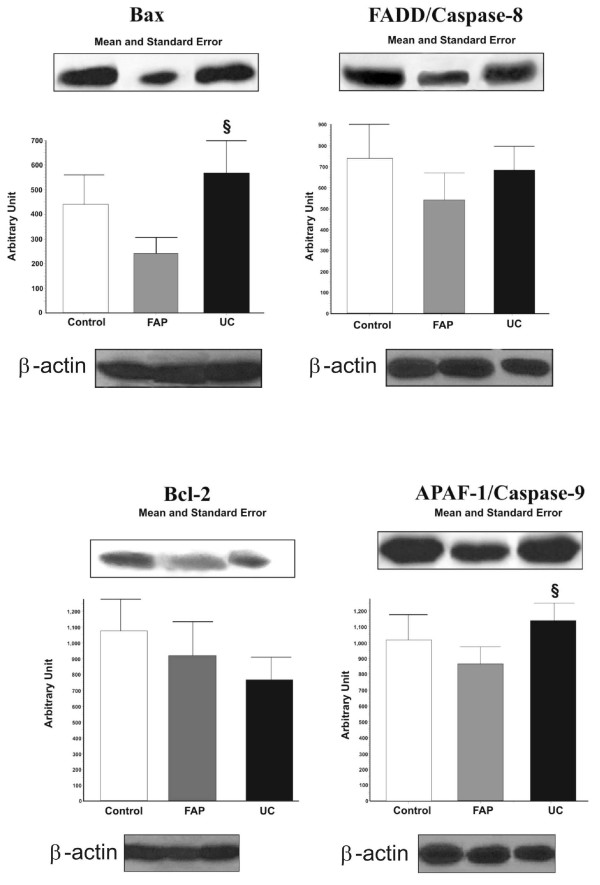
**Representative Western blot analyses and determination of Bax, Bcl-2, FADD - Caspase-8, APAF-1 - Caspase-9 protein expressions in non-inflamed pouches in the Control, FAP and UC groups**. For illustration purpose each line band represents one patient. For all conditions, n = 09, *p < 0.05 vs Control; §p < 0.05 vs FAP.

The determination of proteins expressions are shown in Figure 1.

**Figure 2:**
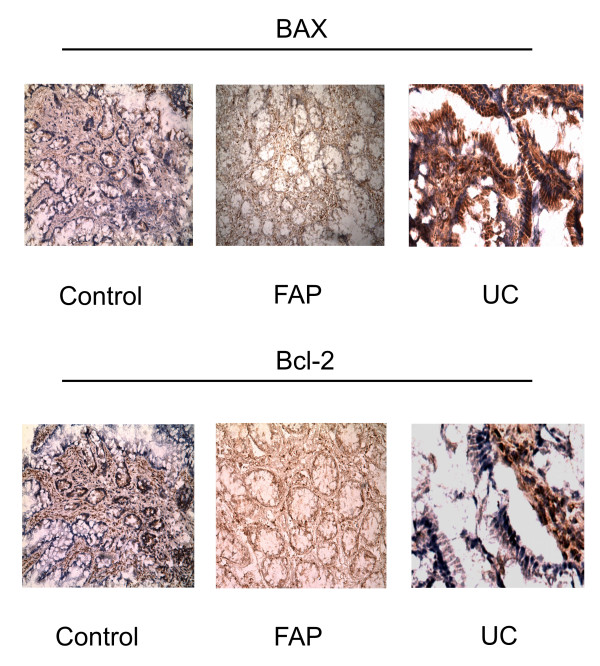
**Immunohistochemistry of ileal pouch sections from UC, FAP and control group immunoreacted for Bax and Bcl-2**. Immunostainig for Bax was intense in UC group. Bcl-2 positive cell among the groups was similar. (200×)

With regard to immunohistochemistry, it showed that immunoreactivity for Bax and Bcl-2 were detected in all groups. Bcl-2 immunostaining pattern was similar among the groups (Figure 2).

## Discussion

Pouchitis is a common complication of total rectocolectomy with ileal pouch-anal anastomosis [25]. The etiology of primary pouchitis remains uncertain and several theories have been suggested like recurrence of UC in ileal pouch. This fact precluded the development of appropriate prophylaxis and treatment.

The fecal stream and stasis play an important part in the pathogenesis of immunological reactions in the ileal pouch, but don't explain the difference in incidence of pouchitis in UC and FAP patients. There were immunological changes in the pouch for at least one year after ileostomy closure in adaptation way after this surgery [26]. Our patients in this study had more than 1 yr of follow-up after ileostomy closure, in order to evaluate apoptosis activity that could lead to pouchitis after this transitional period.

Several cytokines have been reported in ileal pouches, showing that pro-inflammatory cytokines like TNF-α, IL-1β, IL-6, IL-8, IFN-γ are elevated in UC patients, but poorly studied in FAP [4,27-30].

The inflammatory and apoptosis pathways are linked and some pro-inflammatory cytokines, such TNF-α, are evolved in regulation of cell apoptosis. The elevated expression of Fas-Fas-L (CD95-CD95L), a pro-apoptotic member of the TNF-superfamily, has been reported in ileal pouches of patients with UC and a history of pouchitis, but it wasn't compared to FAP patients. It has been related to the role of increased epithelial turn over in the etiology of pouchitis [12]. Indeed, another study reported higher expression of Bad, a potent pro-apoptotic protein of Bcl-2 family, in ileal pouch of UC patients when compared to FAP [13].

This study, we evaluated the expression of pro-apoptotic and anti-apoptotic proteins of Bcl-2 [31,32] and Caspases [33,34] families to evaluate intrinsic and extrinsic pathways of apoptosis in normal ileal pouches. Even in such optimal clinical, endoscopic and histological conditions, the local levels of pro-apoptotics proteins were high in UC patients. Bax, APAF-1 and Caspase-9 expressions were very higher, and FADD, Caspase-8 expressions had a discrete tendency to be more intense in UC patients when compared to FAP. This fact is extremely interesting showing that there is an up-regulating of apoptosis in UC, which could lead or correlate with inflammation tendency in these pouches. It could be due to the fact that both inflammatory and apoptosis pathways are related.

Furthermore, the major pathway of apoptosis in these cases were intrinsic mitochondrial pathway characterized by APAF-1 and Caspase-9 expressions, and it is by according to higher levels of Bad, a member of Bcl-2 family that stays in the mitochondrial membrane, verified in ileal pouches of UC patients in another study [13]. With regard to similar Bcl-2 expression in the different groups, it could mean that all patients were asymptomatic with normal endoscopic and histological features, so there is a balance between pro and anti-apoptotic activities. The increased apoptosis plays an important role in the pathogenesis of pouchitis and probably not a defective of down-regulation promoted by anti-apoptotic proteins. These results were emphasized by results of Bax and Bcl-2 immunohistochemistry.

In the other side, FAP had less expression of pro-apoptotic proteins, thus minor potential cell turn over and it might have a tight connection with primary disease in these patients. Some authors have been reported adenomas in ileal pouch of FAP, more commonly than in UC patients, who have more inflammatory polyps [35-38]. It could be due to low cell turn over that occurs in FAP ileal pouches.

The importance in knowing of the pathways of cell apoptosis in ileal pouch can lead us to understand more about molecular biology involved in pouchitis, and in the primary diseases, FAP and UC.

## Conclusions

In summary, the present study shows that, even under non-inflammatory conditions, patients with UC present higher levels of pro-apoptotic protein in the normal mucosa of pouches. The higher pro-inflammatory cytokines expression in UC, when compared with FAP, verified in the literature, suggests that primary defects of macrophage-lymphocyte regulation, which may coincide with defective regulation of apoptosis, showed in this study, playing an important role in the development of local inflammation in this group of patients. Moreover, we showed a defective regulation of apoptosis in the mucosa of FAP pouches.

## Competing interests

The authors declare that they have no competing interests.

## Authors' contributions

RFL carried out the molecular studies, drafted the manuscript and statistical analysis. MLSA participated in colonoscopy examinations to obtain mucosal biopsies. MM carried out the immunoblotting assays. JJF participated in the design of the study. JCM carried out the Anexin V analysis. LRM carried out the immunohistochemistry procedures. LAV participated in its design and performed the statistical analysis. CSRC participated in its design and coordination, and helped to draft the manuscript. All authors read and approved the final manuscript.
